# Malaria in pregnancy alters L-arginine bioavailability and placental vascular development

**DOI:** 10.1126/scitranslmed.aan6007

**Published:** 2018-03-07

**Authors:** Chloe R. McDonald, Lindsay S. Cahill, Joel L. Gamble, Robyn Elphinstone, Lisa M. Gazdzinski, Kathleen J. Y. Zhong, Adrienne C. Philson, Mwayiwawo Madanitsa, Linda Kalilani-Phiri, Victor Mwapasa, Feiko O. ter Kuile, John G. Sled, Andrea L. Conroy, Kevin C. Kain

**Affiliations:** 1 Institute of Medical Science, University of Toronto, Toronto, Ontario M5S 1A8, Canada; 2 Sandra A. Rotman Laboratories, Sandra Rotman Centre for Global Health, University Health Network-Toronto General Hospital, University of Toronto, Toronto, Ontario M5G 1L7, Canada; 3 Mouse Imaging Centre, Hospital for Sick Children, Toronto, Ontario M5T 3HT, Canada; 4 Department of Medical Biophysics, University of Toronto, Toronto, Ontario M5G 1L7, Canada; 5 Howard Hughes Medical Institute, Department of Neurobiology, Harvard Medical School, Boston, MA 02138, USA; 6 College of Medicine, University of Malawi, P.O. Box 280, Blantyre, Malawi; 7 Liverpool School of Tropical Medicine, Liverpool L35QA, UK; 8 Tropical Disease Unit, Division of Infectious Diseases, Department of Medicine, University of Toronto, Toronto, Ontario M5G 2C4, Canada

## Abstract

Reducing adverse birth outcomes due to malaria in pregnancy (MIP) is a global health priority. However, there are few safe and effective interventions. L-arginine is an essential amino acid in pregnancy and an immediate precursor in the biosynthesis of nitric oxide (NO), but there are limited data on the impact of MIP on NO biogenesis. We hypothesized that hypoarginemia contributes to the pathophysiology of MIP and that L-arginine supplementation would improve birth outcomes. In a prospective study of pregnant Malawian women, we show that MIP was associated with lower concentrations of L- arginine and higher concentrations of endogenous inhibitors of NO biosynthesis, asymmetric and symmetric dimethylarginine, which were associated with adverse birth outcomes. In a model of experimental MIP, L-arginine supplementation in dams improved birth outcomes (decreased stillbirth and increased birth weight) compared with controls. The mechanism of action was via normalized angiogenic pathways and enhanced placental vascular development, as visualized by placental microcomputerized tomography imaging. These data define a role for dysregulation of NO biosynthetic pathways in the pathogenesis of MIP and support the evaluation of interventions to enhance L-arginine bioavailability as strategies to improve birth outcomes.

## INTRODUCTION

An estimated 125 million women become pregnant in malaria-endemic regions every year, with more than 85 million at risk of *Plasmodium falciparum* malaria (*1*–*3*). Pregnant women, particularly first-time mothers, are more likely to be infected with falciparum malaria and to experience complications including maternal anemia, pregnancy loss, and low birth weight (LBW) resulting from small-for-gestational age (SGA) outcomes and/or preterm birth (PTB) (*4*–*7*). Malaria in pregnancy (MIP) leads to the sequestration of malaria-infected red blood cells in the intervillous space of the placenta and the recruitment of mononuclear cells, generating a localized immune response at the maternal-fetal interface (*8*, *9*). MIP-induced immune responses in the placenta can disrupt normal angiogenic processes, resulting in placental insufficiency and the inability of the placenta to support rapid fetal growth in the third trimester, ultimately leading to SGA, PTB, and LBW (*10*).

Despite the negative impact of MIP on global maternal-child health, there are currently limited intervention strategies to improve maternal and neonatal outcomes. Evidence from pre-eclampsia and other causes of adverse pregnancy outcomes suggests that interventions to promote placental angiogenesis may improve birth outcomes (*11*–*16*). L-arginine is an essential amino acid in pregnancy and an immediate precursor in the biosynthesis of nitric oxide (NO) via a family of nitric oxide synthase (NOS) enzymes (*17*–*20*). NO plays a central role in endothelial growth and function as a critical regulator of the vascular endothelial growth factor (VEGF) family of proteins, including placental growth factor (PGF), the angiopoietins (ANG-1 and ANG-2), and their respective soluble receptors (SFLT-1 and STIE-2) (*21*). The VEGF family of proteins is essential for proper placental vascularization, vessel growth, and remodeling throughout pregnancy (*12*, *22*, *23*). NO production increases pro-angiogenic VEGF-A and PGF in human trophoblast cultures, whereas inhibition of NO synthesis results in elevated SFLT-1 and hypertensive responses in pregnant mice (*21*, *24*, *25*). NO also reduces the expression of endothelial adhesion receptors and proinflammatory cytokines, which contribute to increased monocyte accumulation and MIP pathogenesis (*26*, *27*).

There is considerable evidence that reduced bioavailable NO contributes to the pathophysiology of severe malaria (*28*–*30*). Malaria-induced hemolysis depletes L-arginine and NO, contributing to hypoarginemia and impaired NO synthesis (*28*). NO bioavailability is further impaired by the generation of endogenous inhibitors of NO biogenesis, asymmetric dimethylarginine (ADMA) and symmetric dimethylarginine (SDMA). ADMA is a competitive inhibitor of NOS, whereas SDMA enhances inflammation and oxidative stress and, at high concentrations, impairs arginine transport into cells (*31*, *32*). A reduced ratio of L-arginine to ADMA (a measure of L-arginine bioavailability) has been reported in children and adults with severe malaria (*33*, *34*). NO may also be depleted by NO scavenging by cell-free hemoglobin as a result of malaria-induced hemolysis (*33*). In severe malaria, reduced NO bioavailability contributes to endothelial dysfunction and can be reversed in both human infection and experimental models by parenteral L-arginine infusion (*35*, *36*). Pregnancy can also contribute to hypoarginemia because arginine is continuously metabolized to meet the high NO demands required to support placental vascular growth and remodeling (*37*). Moreover, diets deficient in L-arginine are common in low-resource and malaria-endemic regions and may further deplete bioavailable L-arginine during pregnancy (*38*, *39*). We hypothesized that maternal circulating concentrations of L-arginine, ADMA, and SDMA would be altered in women with MIP and that L-arginine supplementation during pregnancy would improve pregnancy outcomes (birth weight and viability) in a preclinical model of MIP by regulating angiogenesis and promoting placental vascular development.

## RESULTS

### MIP is associated with altered ADMA and SDMA concentrations and L-arginine/ADMA ratio

We assessed the L-arginine–NO biosynthetic pathway in plasma samples collected from 384 primigravid Malawian women at enrollment (between 16 and 28 weeks of gestation) in a randomized clinical trial (*40*). Demographic data of the study population are presented in Table 1. Malaria at enrollment was common in this population, with 24% (*n* = 91 of 379) of women positive by microscopy and 57% (*n* = 217 of 381) of women positive by polymerase chain reaction (PCR). Submicroscopic infections were common, with 48.1% (*n* = 137 of 285) of smear-negative women testing positive by PCR. Smear-positive malaria at enrollment was associated with a significant reduction in birth weight compared to women who tested smear-negative for malaria [mean (SD), smear-positive, 2699 (409) g versus smear-negative, 2805 (432) g; *P =* 0.04]. Submicroscopic malaria infections were not associated with changes in birth weight (*P* = 0.73). At delivery, 48% (*n* = 171 of 356) of women had histological evidence of malaria in the placenta (active or past infection). Placental malaria was associated with adverse birth outcomes, with 60.3% of infants born SGA positive for malaria by histology compared to 44.6% of appropriate-for-gestational age (AGA) infants (*P* = 0.01). There was no difference in the proportion of women with histologically defined placental malaria according to treatment arm (*P* = 0.48), and treatment arm was not associated with adverse birth outcomes (LBW, PTB, SGA, *P* > 0.05 for all) or birth weight {mean (SD), intermittent-presumptive treatment in pregnancy [sulfadoxine pyrimethamine (IPTp-SP)], 2773 (461) g versus intermittent screening and treatment in pregnancy [dihydroartemisinin-piperaquine (ISTp-DP)], 2790 (388) g; *P* = 0.69}.

**Table 1:** **Characteristics of pregnant women enrolled in a prospective cohort study in southern Malawi.** Data are presented as means (SD) or n (%), unless otherwise indicated. RR, relative risk; CI, confidence interval; BMI, body mass index; MUAC, middle-upper arm circumference; ADMA, asymmetric dimethylarginine; SDMA, symmetric dimethylarginine.

	*n*	Population (*n* = 384)	Longitudinal population (*n* = 94)	Bivariate analysis			Multivariate analysis*	
				AGA (*n* = 302)	SGA (*n* = 82)	*P*	RR (95% CI)	*P*
**Maternal characteristics**								
Age (years)	384	18.0 (1.8)	18.0 (1.8)	18.0 (1.8)	18.1 (1.7)	0.840	1.05 (0.94–1.16)	0.407
Height (cm)	384	153 (4.3)	154 (4.8)	153 (4.4)	152 (4.1)	0.216	–	
Weight (kg)	381	53.4 (6.3)	54.1 (7.4)	53.8 (6.3)	51.8 (6.2)	0.011	–	
BMI (kg/m)†	381	22.8 (2.6)	22.8 (3.0)	22.9 (2.6)	22.2 (2.6)	0.043	0.93 (0.86–1.01)	0.089
MUAC (cm)	237	23.5 (1.6)	23.8 (1.8)	23.5 (1.6)	23.4 (1.6)	0.680	—	
Socioeconomic status† (tertile)								
1	381	135 (35.4)	34 (36.2)	110 (36.8)	25 (30.5)	0.148	Reference	
2		111 (29.1)	20 (21.3)	80 (26.8)	31 (37.8)		1.65 (1.04-2.64)	0.035
3		135 (35.4)	24 (25.5)	66 (22.1)	18 (22.0)			
Education status†(tertile)								
1	381	68 (17.9)	14 (14.9)	57 (19.1)	11 (13.4)	0.471	—	
2		229 (60.1)	56 (59.6)	176 (58.9)	53 (64.6)			
3		84 (22.1)	24 (25.5)	66 (22.1)	18 (22.0)			
Treatment arm (IPTp), n (%)	382	199 (51.8)	40 (42.6)	158 (52.3)	41 (50.0)	0.710	1.05 (0.71-1.54)	0.808
Hemoglobin (g/dl)	376	10.3 (1.4)	10.0 (1.4)	10.3 (1.4)	10.1 (1.5)	0.272	—	
Gestational age at enrollment (weeks)	384	20.4 (3.1)	20.9 (3.4)	20.6 (3.0)	19.7 (3.2)	0.032	0.93 (0.86-0.99)	0.036
**Malaria at enrollment**								
Microscopy, *n* (%)	379	91 (24.0)	21 (23.1)	71 (23.8)	20 (24.7)	0.871	0.96 (0.60-1.55)	0.879
PCR, n (%)	381	217 (57.0)	57 (61.3)	171 (57.2)	46 (56.1)	0.859	—	
Submicroscopic‡, *n* (%)	285	137 (48.1)	37 (53.6)	108 (48.2)	29 (47.5)	0.926	—	
**Arginine pathway at enrollment**								
L-arginine (µM)	379	32.3 (19.6)	36.8 (17.0)	31.1 (19.6)	32.9 (19.6)	0.750	—	
ADMA (µM)	379	0.44 (0.08)	0.49 (1.0)	0.44 (0.08)	0.46 (0.10)	0.030	21.2 (2.27-197.9)	0.007
SDMA (µM)	379	0.37 (0.07)	0.40 (0.07)	0.37 (0.07)	0.38 (0.07)	0.632	—	
L-arginine/ADMA	379	75.3 (49.4)	79.8 (44.0)	75.1 (48.3)	75.9 (53.7)	0.902	—	
**Birth outcomes**								
Infant sex (female)	384	188 (49.0)	42 (44.7)	145 (48.0)	43 (52.4)	0.477	—	
Birth weight (g)	384	2781 (427)	2759 (456)	2859 (428)	2493 (272)	<0.0001	—	
Gestational age at delivery (weeks)	384	38.1 (2.3)	38.1 (2.2)	37.7 (2.3)	39.2 (1.7)	<0.0001	—	
**Placental malaria**								
Histology	356	171 (48.0)	45 (51.1)	124 (44.6)	47 (60.3)	0.014	—	

* Log-binomial model.

† Median (interquartile range). Bivariate analysis by Student’s t test or Wilcoxon rank sum test.

‡ Submicroscopic malaria is defined as polymerase chain reaction (PCR)–positive and microscopy-negative. Participants who were positive by microscopy were excluded (*n* = 91, where *n* = 15 were positive by microscopy but negative by PCR), as were those who were missing microscopy results (*n* = 5) or missing PCR results (*n* = 4).

Women with smear-positive malaria at enrollment had higher concentrations of SDMA and ADMA than women who were smear-negative (Table 2). There was no association between plasma L-arginine concentrations and microscopy-defined malaria at enrollment. Women with PCR-defined malaria or submicroscopic malaria had significantly higher concentrations of SDMA (*P* < 0.001) and ADMA (*P* < 0.05) and a lower L-arginine/ADMA ratio (*P* < 0.01) than women with PCR-negative malaria (Table 2). After adjustment for maternal age and gestational age at enrollment, a one-unit increase in ADMA or SDMA was associated with a relative risk [95% confidence interval (CI)] of PCR-defined malaria of 6.40 (3.72 to 11.04) and 3.94 (1.61 to 9.66), respectively (Table 2). This effect was strongest in the 48.1% of women with submicroscopic infections, for whom a one-unit increase in ADMA or SDMA was associated with a 10.29 (5.04 to 21.02) and 8.44 (1.77 to 40.29) increased relative risk of submicroscopic malaria, respectively (Table 2).

**Table 2:** **Association between the nitric oxide biosynthetic pathway and malaria at enrollment.** Malaria at enrollment is determined in maternal blood at enrollment, data are presented as means (SD) and analyzed using Student’s t test, and log-binomial regression was used to obtain relative risk and corresponding 95% confidence intervals adjusting for maternal age and gestational age at enrollment.

	Malaria-negative	Malaria-positive	*P*	Unadjusted model, RR (95% CI)	Adjusted model, RR (95% CI)
**Microscopy-defined malaria**	***n* = 285**	***n* = 89**			
L-arginine (µM)	32.3 (19.2)	32.8 (21.2)	0.926	1.00 (0.99-1.01)	1.00 (0.99-1.01)
ADMA (µM)	0.44 (0.09)	0.46 (0.08)	0.044	6.72 (1.05-43.10)	6.18 (0.93-40.96)
SDMA (µM)	0.37 (0.07)	0.39 (0.06)	0.029	12.00 (1.24-116.16)	**24.23 (2.09-280.90)**
L-arginine/ADMA	76.0 (48.4)	73.6 (53.5)	0.698	1.00 (1.00-1.00)	1.00 (1.00-1.00)
**PCR-defined malaria**	***n* = 163**	***n* = 214**			
L-arginine (µM)	33.9 (21.2)	31.1 (18.2)	0.160	1.00 (0.99-1.00)	0.996 (0.987-1.005)
ADMA (µM)	0.42 (0.08)	0.46 (0.08)	<0.0001	6.74 (4.13-11.01)	**6.40 (3.72-11.04)**
SDMA (µM)	0.36 (0.07)	0.38 (0.07)	0.009	4.24 (1.49-12.08)	**3.94 (1.61-9.66)**
L-arginine/ADMA	83.7 (56.0)	69.0 (43.0)	0.004	0.997 (0.994-0.999)	**0.997 (0.994-0.999)**
Submicroscopic malaria*	***n* = 147**	***n* = 136**			
L-arginine (µM)	34.0 (21.7)	30.6 (16.1)	0.143	0.995 (0.988-1.002)	0.993 (0.982-1.005)
ADMA (µM)	0.41 (0.08)	0.47 (0.09)	<0.0001	11.60 (6.03-22.32)	**10.29 (5.04-21.02)**
SDMA (µM)	0.36 (0.07)	0.38 (0.07)	0.037	6.02 (1.21-29.90)	**8.44 (1.77-40.29)**
L-arginine/ADMA	84.7 (57.8)	66.8 (33.7)	0.002	0.995 (0.992-0.999)	**0.995 (0.992-0.999)**

* Submicroscopic malaria is defined as PCR-positive and microscopy-negative. Women were excluded if they were missing microscopy results (*n* = 5) or PCR results (*n* = 4) or were microscopy-positive (*n* = 91)

### Altered L-arginine concentration is associated with maternal nutritional status

Because L-arginine is a conditionally essential amino acid obtained through the consumption of dietary protein, we explored whether concentrations of L-arginine, SDMA, and ADMA were related to maternal nutritional status [maternal body mass index (BMI) or middle-upper arm circumference (MUAC)] by regression analysis (Table 3). An increase in L-arginine or the L-arginine/ADMA ratio was positively associated with MUAC after adjustment for maternal age and gestational age at enrollment (*P* = 0.005 and *P* = 0.003, respectively; Table 3), suggesting that higher L-arginine concentrations are associated with improved nutritional status. There was a strong negative relationship between ADMA and SDMA and maternal hemoglobin after adjustment for maternal age and gestational age at enrollment (*P* < 0.0001 and *P* = 0.001, respectively; Table 3).

**Table 3:** **Regression analysis examining the nitric oxide biosynthetic pathway and maternal nutritional status.** Linear regression analysis was used, adjusting for maternal age and gestational age at enrollment.

	BMI (kg/m^2^)		MUAC (cm)		Hemoglobin (g/dl)	
	Unadjusted model, β (SE)	Adjusted model, β (SE)	Unadjusted model, β (SE)	Adjusted model, β (SE)	Unadjusted model, β (SE)	Adjusted model, β (SE)
L-arginine (µM)	0.005 (0.007), *P* = 0.432	0.004 (0.007), *P* = 0.523	0.018 (0.008), *P* = 0.021	0.024 (0.009), *P* = 0.005	0.001 (0.004), *P* = 0.818	0.001 (0.004), *P* = 0.842
ADMA (µM)	-1.23 (1.56), *P* = 0.431	-1.07 (1.56), *P* = 0.495	-1.42 (1.20), *P* = 0.241	-1.09 (1.23), *P* = 0.373	-6.06 (0.82), *P* < 0.0001	-6.01 (0.83), *P* < 0.0001
SDMA (µM)	-1.01 (1.90), *P* = 0.597	-2.63 (1.95), *P* = 0.180	-0.64 (1.50), *P* = 0.669	-0.304 (1.57), *P* = 0.845	-3.80 (1.06), *P* < 0.0001	-3.80 (1.10), *P* = 0.001
L-arginine/ADMA	0.003 (0.003), *P* = 0.255	0.002 (0.003), *P* = 0.357	0.008 (0.003), *P* = 0.010	0.010 (0.003), *P* = 0.003	0.003 (0.001), *P* = 0.060	0.003 (0.001), *P* = 0.072

### Increased ADMA at enrollment is associated with adverse birth outcomes

We investigated the association between the L-arginine pathway and adverse birth outcomes. A total of 167 (43.5%) women had an adverse birth outcome consisting of PTB or SGA. Adverse birth outcomes (PTB or SGA as a composite outcome) were associated with increased ADMA at enrollment compared to normal birth outcome [mean (SD), term/AGA, 0.43 µM (0.07); adverse birth outcome, 0.46 µ M (0.09); *P* = 0.007 (Student’s *t* test)]. Using log-binomial regression, ADMA was associated with an increased relative risk of having an SGA infant [adjusted relative risk, 21.2 (95% CI, 2.27 to 197.9); *P* = 0.007] after adjustment for maternal age, gestational age at enrollment, BMI, socioeconomic status, smear-positive malaria at enrollment, and treatment group (Table 1).

### MIP is associated with lower concentrations of L-arginine and higher concentrations of ADMA across pregnancy

**Fig. 1:**
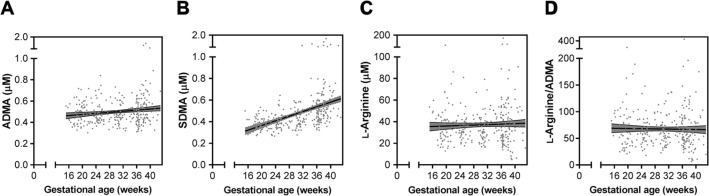
**L-arginine, ADMA, and SDMA change longitudinally over the course of pregnancy.** Concentrations of L-arginine, ADMA, and SDMA measured by mass spectrometry in *n* = 603 plasma samples from the study cohort of pregnant women (*n* = 94) beginning at 16 weeks of gestation. (**A** to **D**) Longitudinal assessment of changes during pregnancy in ADMA (*P* = 0.01) (A), SDMA (*P* < 0.0001) (B), L-arginine (*P* > 0.05) (C), and L-arginine/ADMA (*P* > 0.05) (D) from 16 weeks of gestation to delivery by linear regression.

To evaluate the kinetics of the L-arginine pathway across pregnancy, we quantified longitudinal concentrations of L-arginine, ADMA, and SDMA in the plasma of 94 of the 384 women included in this study, who had between two and five samples collected before delivery (mean of 3.3 visits; *n* = 603 samples tested). We observed an increase in plasma concentrations of ADMA (Fig. 1A) and SDMA (Fig. 1B) over the course of pregnancy (*P* = 0.01 and *P* < 0.0001, respectively, linear regression of biomarker concentrations by gestational age). There was no change in concentrations of L-arginine (Fig. 1C) or L-arginine/ADMA (Fig. 1D) during pregnancy (*P* > 0.05 for both outcomes).

**Fig. 2:**
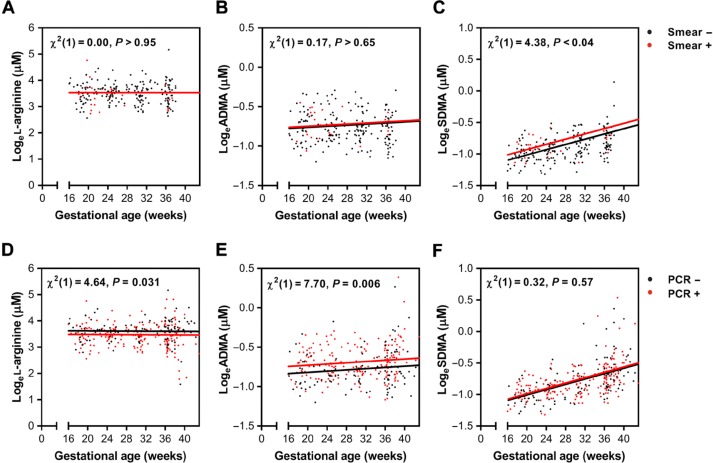
**Malaria at enrollment is associated with altered nitric oxide biosynthesis during pregnancy.** (**A** to **F**) Individual data points colored according to malaria status by microscopy for concentrations of L-arginine (*P* > 0.95) (A), ADMA (*P* > 0.65) (B), and SDMA (*P* < 0.04) (C) or malaria status by PCR for L-arginine (*P* = 0.031) (D), ADMA (*P* = 0.006) (E), and SDMA (*P* = 0.57) (F). The overlaid regression lines are from linear mixed-effects models fitted for a subject with average values (conditional on fixed effects only).

At enrollment, 61.3% (*n* = 57 of 93) of these women were positive for malaria by PCR and 23.1% (*n* = 21 of 91) were positive by microscopy. Of the infections detected, 53.6% (*n* = 37 of 69) were submicroscopic (PCR-positive and microscopy-negative), and 51.1% (*n* = 45 of 88) of women had histologically defined placental malaria at delivery (Table 1). At enrollment, ADMA concentrations were higher than SDMA concentrations, but SDMA increased more than ADMA over gestation, and SDMA concentrations surpassed those of ADMA by 36 weeks of gestation (Fig. 1). Using linear mixed-effects modeling, we found that malaria detected by microscopy at enrollment was associated with significantly higher concentrations of SDMA during pregnancy [χ^2^(1) = 4.38, *P* < 0.04]; however, there were no differences in concentrations of ADMA [χ ^2^(1) = 0.17, *P* > 0.65] or L-arginine [χ ^2^(1) = 0.00, *P* > 0.95] during pregnancy based on malaria status at enrollment (Fig. 2, A to C). Conversely, PCR-defined malaria at enrollment was associated with higher ADMA over the course of gestation [χ ^2^(1) = 7.70, *P* = 0.006] and lower L-arginine [χ ^2^(1) = 4.64, *P* = 0.031] (Fig. 2, D to F).

**Fig. 3:**
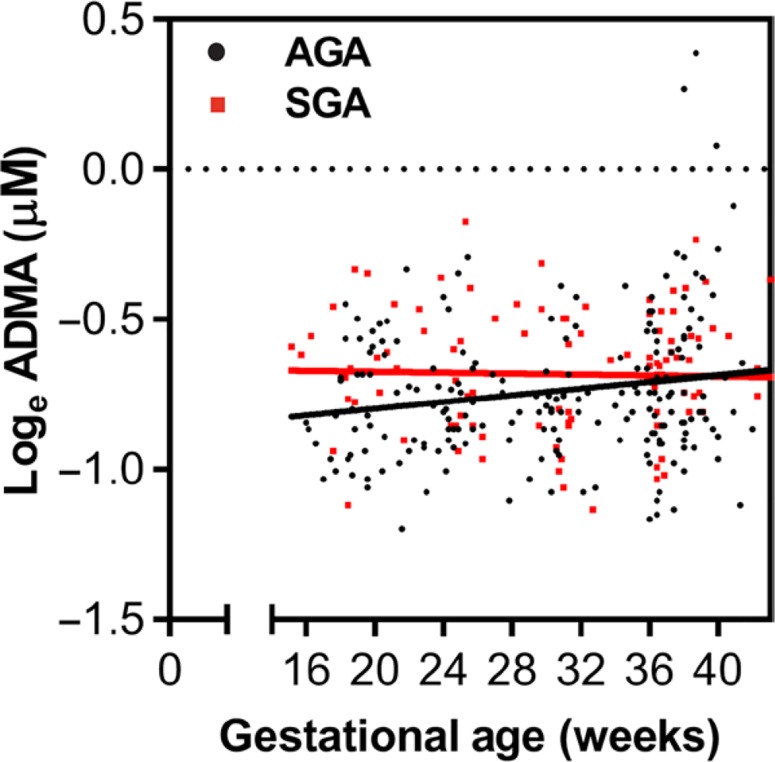
**Increased ADMA in pregnancy is associated with SGA birth outcomes.** Those who went on to have SGA births had higher concentrations of ADMA than those who later had AGA births [χ^2^(2) = 8.76, *P* < 0.02]. Individual data points are colored according to SGA or AGA status with overlaid regression lines from linear mixed-effects models, fitted for a subject with average values (conditional on fixed effects only).

Because increased ADMA at enrollment was associated with increased relative risk of SGA, we explored this relationship further, comparing ADMA concentrations over the course of pregnancy (Fig. 3). We used linear mixed-effects modeling to evaluate the relationship between the ADMA concentrations and the SGA outcome, adjusting for gestational age, maternal age, BMI, malaria status, socioeconomic status, and the interaction between treatment arm and gestational age. Those who subsequently delivered SGA newborns had significantly higher concentrations of ADMA during pregnancy than those who later had AGA births [χ ^2^(2) = 8.76, *P* < 0.02]. The groups converged over time, as demonstrated by the addition of the interaction term between SGA and gestational age [χ ^2^(1) = 4.62, *P* < 0.04; table S1]. Thus, increased ADMA early in pregnancy (reflecting reduced NO biosynthesis) is associated with SGA, but the effect diminishes over the course of gestation, and those with lower baseline concentrations of ADMA show increases later in pregnancy.

### Dietary L-arginine supplementation improves fetal weight and fetal viability in an experimental model of MIP

**Fig. 4:**
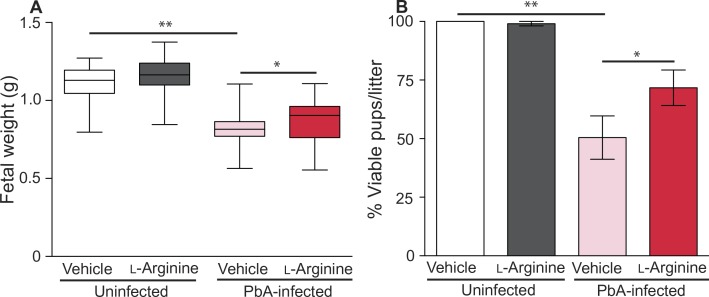
**Dietary L-arginine supplementation improves fetal outcomes in experimental malaria in pregnancy.** (**A**) Fetal weight (in grams) in uninfected vehicle control–treated litters (*n* = 22), uninfected L-arginine–supplemented litters (*n* = 37), malaria [*Plasmodium berghei* ANKA (PbA)]–infected vehicle control–treated litters (*n* = 26), and malaria (PbA)–infected L-arginine–supplemented litters (*n* = 36). Box plots depict median, and interquartile range with whiskers depicts maximum and minimum values. (**B**) Percentage of viable pups per litter in uninfected vehicle control–treated litters (*n* = 22), uninfected L-arginine–supplemented litters (*n* = 37), malaria (PbA)–infected vehicle control–treated litters (*n* = 26), and malaria (PbA)–infected L-arginine–supplemented litters (*n* = 36). The figure depicts mean ± SD.Results of independent samples *t* test (fetal weight) and χ2 test (viability); *P < 0.05 and **P < 0.001.

On the basis of the findings of altered pathways of L-arginine and NO biosynthesis in humans, we next explored the mechanism and interventions in an experimental MIP (EMIP) mouse model. EMIP recapitulates several features of human MIP, including placental parasite accumulation, damage to the syncytiotrophoblast, an LBW phenotype (*P* < 0.001; Fig. 4A), and reduced fetal viability (a surrogate for stillbirth) assessed at gestational day 19 (G19) (*P* < 0.001; Fig. 4B) (*10*, *41*). We used the EMIP model to examine the impact of dietary L-arginine on birth outcomes. L-arginine supplementation to dams did not influence maternal peripheral parasite densities (G19) or litter size (table S2). L-arginine supplementation did not alter birth weight or fetal viability in offspring from uninfected, control litters (Fig. 4, A and B). However, in malaria-infected dams, L-arginine supplementation increased fetal weight (*P* < 0.05; Fig. 4A and table S3) and increased the number of viable pups per litter (*P* < 0.05; Fig. 4B and table S4). No differences were observed in placental weight between treatment groups (table S3). EMIP was associated with a 24.62-fold increased relative risk of delivering a nonviable pup (95% CI, 12.90 to 46.98; *P* < 0.001). L-arginine supplementation during EMIP was associated with a 2.5-fold decrease in the relative risk of delivering a nonviable pup [reduced from 24.62 to 9.65 (5.32 to 17.51); *P* < 0.05; table S4].

### EMIP decreases circulating L-arginine

**Fig. 5:**
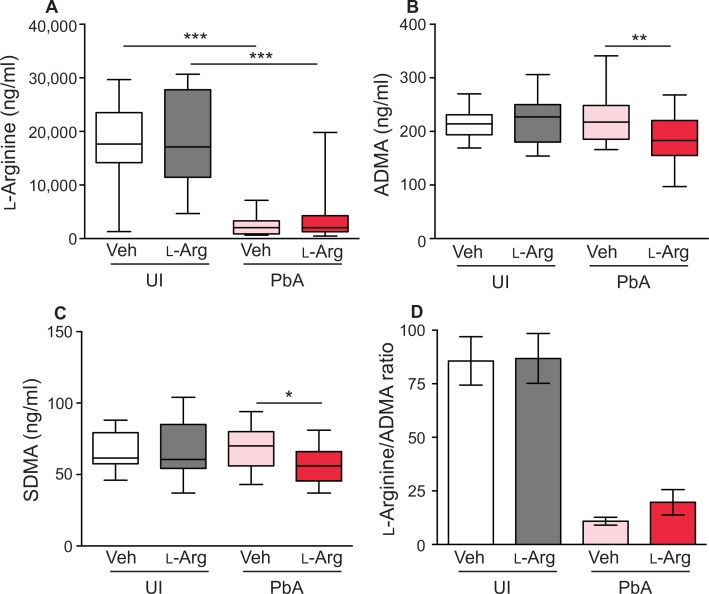
**L-arginine, ADMA, and SDMA concentrations are altered in experimental malaria in pregnancy.** (**A**) Malaria infection is associated with decreased L-arginine serum concentrations, as measured by mass spectrometry. (**B** and **C**) Malaria-infected dams receiving L-arginine supplementation show reduced serum concentrations of ADMA (B) and SDMA (C). Box plots depict median, and interquartile range with whiskers depicts maximum and minimum values. (**D**) L-arginine/ADMA ratio in mice with or without malaria infection and/or L-arginine supplementation. Malaria-infected vehicle control–treated (Veh) (*n* = 18) and malaria-infected (PbA) L-arginine–treated (L-Arg) dams (*n* = 17) were compared with uninfected (UI) vehicle control (*n* = 14) and uninfected L-arginine–treated dams (*n* = 16). Figures depict mean ± SD. Results of one-way analysis of variance (ANOVA) and Tukey post test; *P < 0.05, **P < 0.01, and ***P < 0.001.

We performed mass spectrometry on serum collected at G19 to quantify circulating concentrations of L-arginine, ADMA, and SDMA in malaria-infected and uninfected pregnant dams. At G19 (day 6 of infection), concentrations of L-arginine were significantly reduced in malaria-infected dams (*P* < 0.001; Fig. 5A). Malaria-infected dams receiving L-arginine supplementation showed reduced serum ADMA (*P* < 0.01; Fig. 5B) and SDMA (*P* < 0.05; Fig. 5C), although there was no significant difference in L-arginine/ADMA ratio (*P* > 0.05; Fig. 5D) compared with the control group.

### L-arginine supplementation in EMIP alters inflammatory and angiogenic mediators in the placenta

**Fig. 6:**
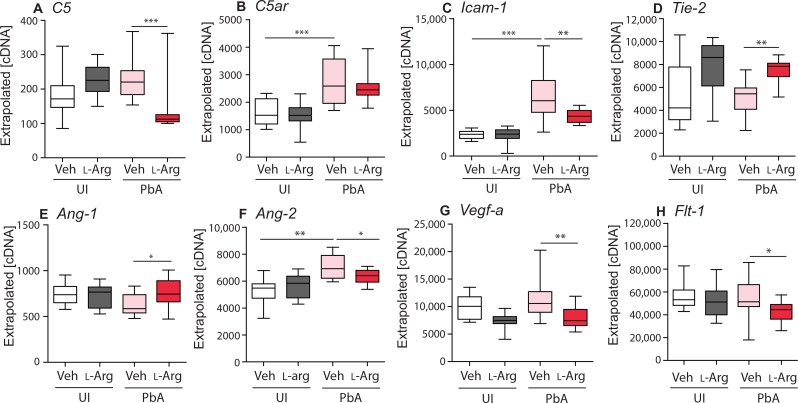
**Malaria and L-arginine induce changes in the expression of inflammatory and angiogenic mediators in placental tissue by reverse transcription PCR**. (A to H) *C5* (A), *C5ar* (B), *Icam-1* (C), *Tie-2* (D), *Ang-1* (E), *Ang-2* (F), *Vegf-a* (G), and *Flt-1* (VEGF receptor) (H) in uninfected (UI) vehicle control–treated (Veh) dams (*n* = 12), uninfected (UI) L-arginine–treated (L-Arg) dams (*n* = 12), malaria-infected (PbA) vehicle control–treated dams (*n* = 12), and malaria-infected L-arginine–treated dams (*n* = 12). Box plots depict median, and interquartile range with whiskers depicts maximum and minimum values. Results of one-way ANOVA and Tukey post test; *P < 0.05, **P < 0.01, and ***P < 0.001.

We hypothesized that L-arginine supplementation increases fetal weight and viability by reducing malaria-induced inflammation in the placenta and by promoting the placental vascular development and remodeling required for healthy pregnancy outcomes. Therefore, we examined the expression of inflammatory and angiogenic factors in placental tissue from viable pups collected at G19 (Fig. 6). EMIP resulted in increased placental expression of the proinflammatory C5a receptor (*C5ar*; *P* < 0.001; Fig. 6B), *Icam-1* (*P* < 0.001; Fig. 6C), and pro-angiogenic *Ang-2* (*P* < 0.01; Fig. 6F). L-arginine supplementation did not alter gene expression in placental tissue from uninfected dams parasite accumulation, damage to the syncytiotrophoblast, an LBW phenotype (*P* < 0.001; Fig. 4A), and reduced fetal viability (a surrogate for stillbirth) assessed at gestational day 19 (G19) (*P* < 0.001; Fig. 4B) (*10*, *41*). We used the EMIP model to examine the impact of dietary L-arginine on birth outcomes. L-arginine supplementation to dams did not influence maternal peripheral parasite densities (G19) or litter size (table S2). L-arginine supplementation did not alter birth weight or fetal viability in offspring from uninfected, control litters (Fig. 4, A and B). However, in malaria-infected dams, L-arginine supplementation increased fetal weight (*P* < 0.05; Fig. 4A and table S3) and increased the number of viable pups per litter (*P* < 0.05; Fig. 4B and table S4). No differences were observed in placental weight between treatment groups (table S3). EMIP was associated with a 24.62-fold increased relative risk of delivering a nonviable pup (95% CI, compared to control uninfected dams. In malaria-infected dams supplemented with L-arginine, we observed reduced gene expression of inflammation-related proteins *C5* (*P* < 0.001; Fig. 6A) and *Icam-1* (*P* < 0.01; Fig. 6C). L-arginine supplementation in malaria-infected dams also resulted in changes to angiogenic mediators, with an up-regulation of *Tie-2* (*P* < 0.01; Fig. 6D) and *Ang-1* (*P* < 0.05; Fig. 6E) and a down-regulation of *Ang-2* (*P* < 0.05; Fig. 6F). In addition, there was reduced expression of the pro-angiogenic factor *Vegf-a* (*P* < 0.01; Fig. 6G) and its negative regulator *Flt-1* (*P* < 0.05; Fig. 6H) in placental tissue from malaria-infected dams receiving L-arginine supplementation compared to malaria-infected control dams. Overall, L-arginine supplementation during EMIP resulted in a more balanced angiogenic response expected to favor vessel remodeling in the placenta.

### L-arginine supplementation during EMIP increases placental vascular development and remodeling

**Fig. 7:**
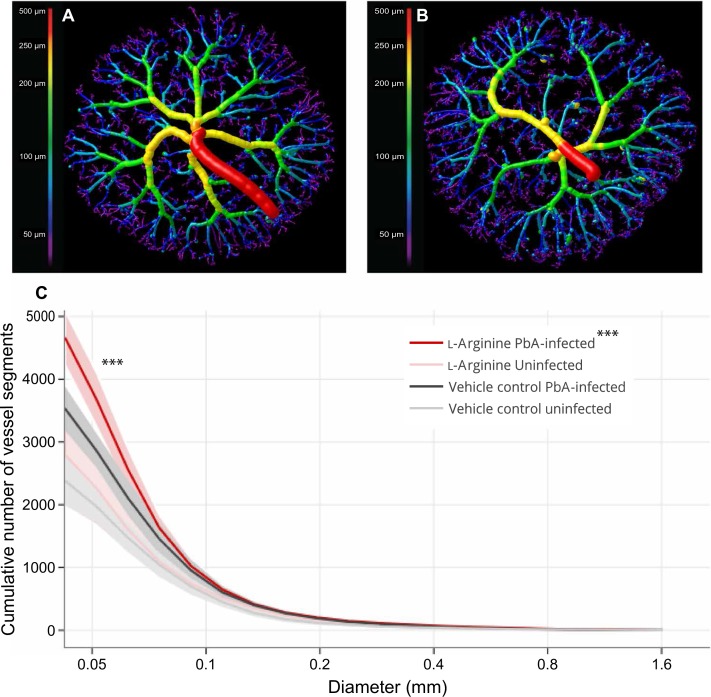
**L-arginine supplementation increases the number of small vessels in placentas from malaria-infected litters.** (**A** and **B**) Representative micro-computed tomography images of fetoplacental arterial vasculature at gestational day 18 in placentas from malaria-infected vehicle control–treated (A) and L-arginine–treated (B) mice color-coded by vessel diameter. (**C**) Cumulative distribution of vessel diameters in placentas from uninfected vehicle control–treated (*n* = 7), uninfected L-arginine–treated (*n* = 7), malaria-infected vehicle control–treated (*n* = 8), and malaria infected L-arginine–treated (*n* = 7) litters. Cumulative vessel segments are depicted as median and SEM of vessels larger than the threshold diameter (0.035 mm) with results of two-way ANOVA and Dunn’s multiple comparison post hoc test; ***P < 0.001.

To examine whether changes observed in placental tissue expression of angiogenic factors associated with L-arginine supplementation were related to functional changes in placental vascular development, we performed micro-computed tomography (micro-CT) imaging of placentas collected before the onset of the LBW and stillbirth phenotypes (*10*). In light of previously reported malaria-induced changes in placental vascular development in association with enhanced C5a-C5aR signaling (*10*), we hypothesized that L-arginine supplementation, similar to C5aR blockade, would increase placental vascularization and improve birth outcomes. In uninfected litters supplemented with L-arginine, we did not observe differences in placental vascularization compared to uninfected control litters (Fig. 7). In contrast, malaria- infected dams receiving L-arginine had an increased total number of placental vessel segments compared with L-arginine–treated uninfected controls (*P* = 0.02; Fig. 7, A and B). Placentas from L-arginine–treated malaria-infected dams showed higher numbers of vessel segments in vessels with a diameter of <50 µ m compared with placentas from vehicle control–treated malaria-infected litters (*P* < 0.001; Fig. 7C).

### L-arginine supplementation increases fetal weight and viability in the context of an L-arginine–deficient diet

**Fig. 8:**
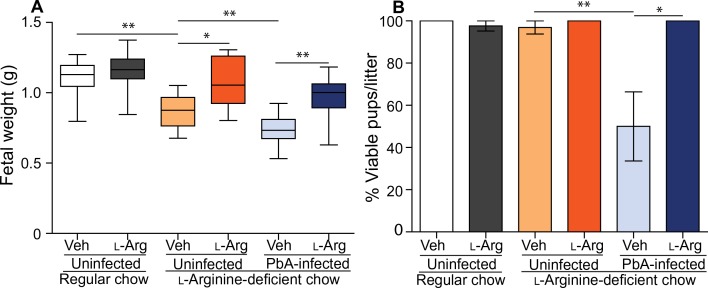
**Dietary L-arginine supplementation improves birth outcomes in malaria-infected dams receiving L-arginine–deficient chow**. (**A**) Fetal weight (in grams) of uninfected vehicle control–treated (Veh) litters (*n* = 22) and uninfected L-arginine–supplemented (l-Arg) litters on regular chow (*n* = 36), as well as uninfected vehicle control–treated litters (*n* = 10), uninfected L-arginine–supplemented litters (*n* = 10), malaria (PbA)–infected vehicle control–treated litters (*n* = 12), and malaria (PbA)–infected L-arginine–supplemented litters (*n* = 11) on L-arginine– deficient chow. Box plots depict median, and interquartile range with whiskers depicts maximum and minimum values. (**B**) Percentages of viable pups per litter in the groups of mice shown in (A). The figure depicts mean ± SD. Results of one-way ANOVA and Tukey post test; *P < 0.05 and **P < 0.01.

Pregnant women in malaria-endemic areas are particularly vulnerable to hypoarginemia due to diets that are relatively deficient in L-arginine because staple foodstuffs (maize, plantains, yams, and cassava) are low in dietary L-arginine (*42*). Therefore, we modeled this scenario by placing dams on an L-arginine–deficient diet and hypothesized that this would increase the impact of L-arginine supplementation on birth outcomes in EMIP. Compared with controls receiving regular chow, offspring of uninfected dams on the L-arginine–deficient chow had lower birth weight (*P* < 0.01; Fig. 8A), and supplementation with L-arginine reversed the LBW phenotype (*P* > 0.05; Fig. 8A). Litters born to malaria-infected dams on the deficient chow that were receiving L-arginine supplementation had increased birth weight (*P* < 0.01; Fig. 8A) and fetal viability (*P* < 0.05; Fig. 8B) compared with infected control litters on the deficient chow.

## DISCUSSION

MIP is a leading global cause of maternal morbidity and adverse pregnancy outcomes. The World Health Organization recommends the use of intermittent presumptive treatment and insecticide-treated nets for the prevention of MIP; however, escalating drug and insecticide resistance threaten this approach (*1*, *40*). We also lack effective and safe interventions to prevent or reduce malaria-associated placental pathology that directly contributes to poor birth outcomes, especially in early pregnancy. Here, we investigated the L-arginine– NO biosynthetic pathway in the pathogenesis of MIP and provide several lines of evidence supporting this axis as a potential therapeutic target. First, in a prospective study of pregnant women in Malawi, we identified MIP-related decreases in circulating concentrations of L-arginine and increases in inhibitors of NO biosynthesis, ADMA and SDMA, and their association with poor birth outcomes. In an experimental model of MIP, we corroborated the human data showing that alterations in NO biogenesis were associated with adverse birth outcomes. We then used this preclinical model to explore the mechanism and interventions and show that L-arginine dietary supplementation improved fetal weight and markedly reduced stillbirth. The effect of supplementation on fetal weight was enhanced when dams were placed on an L-arginine–deficient diet, simulating diets prevalent in low-resource settings. The mechanism of L-arginine action involved reduced expression of placental inflammatory factors, normalized expression contribute to sustained changes in NO bioavailability over pregnancy. Consistent with this hypothesis, increased ADMA between weeks 16 and 28 of pregnancy was associated with impaired fetal growth, and this change was evident across pregnancy. Our results support a mechanistic role for altered L-arginine–NO biosynthesis and related placental insufficiency in malaria-induced SGA outcomes. However, other pathways may also contribute, including those that regulate the nutrient transport across the placenta (*48*).

Collectively, our results suggest that targeting NO biosynthesis in MIP may be an effective intervention to improve birth outcome. In support of this hypothesis, dietary L-arginine supplementation in the EMIP model normalized angiogenic and inflammatory pathways and enhanced placental vascular development. We observed reduced concentrations of circulating L-arginine in both treated and untreated malaria-infected dams. Although L-arginine supplementation did not increase L-arginine in plasma, it was associated with reduced ADMA and SDMA concentrations compared to malaria-infected untreated dams. Plasma samples were collected via cardiac puncture at G19, when dams are ill because of malaria infection and drink less water, and therefore may ingest less L-arginine. Because L-arginine supplementation reduced the circulating inhibitors of NO biosynthesis, ADMA and SDMA, NO bioavailability may have increased even in the absence of increased L-arginine concentrations. of angiogenic mediators, and a corresponding increase in placental vascular development, as evidenced by micro-CT imaging.

NO regulates essential mediators of placental vasculogenesis and angiogenesis, including the VEGF-A and the angiopoietin–TIE-2 pathways, and is critical to implantation, trophoblast invasion, and placental and embryo development (*17*, *21*, *43*, *44*). NO increases the expression of ANG-1 in endothelial cells, and NO production is necessary for VEGF-A–mediated angiogenesis (*43*, *45*). Pathological pregnancy outcomes, including pre-eclampsia, fetal growth restriction, and resulting SGA, have been linked to L-arginine deficiency, reduced NO bioavailability, and oxidative stress (*17*, *46*, *47*). In this prospective study of pregnant Malawian women, we demonstrated that MIP affects NO biogenesis by increasing concentrations of endogenous inhibitors, ADMA and SDMA, and decreasing L-arginine, resulting in decreased L-arginine bioavailability (a reduced L-arginine/ ADMA ratio) and conditions that enhance inflammation while impairing L-arginine bioavailability and intracellular influx (*30*–*32*, *37*, *46*). The impact of malaria on the L-arginine pathway was most evident in PCR-detectable infections at enrollment (16 to 28 weeks of pregnancy) and affected more than half of the women enrolled in this study. These changes occurred relatively early in gestation and could Our findings are supported by previous studies reporting reduced concentrations of ADMA in association with L-arginine supplementation (*49*, *50*). Although the mechanism by which L-arginine reduces ADMA and SDMA is unknown, we speculate that L-arginine supplementation may decrease oxidative stress, the condition under which these endogenous inhibitors are generated (*49*, *51*).

Previous mechanistic studies in preclinical models have shown that MIP alters placental vascular development and results in increased placental arterial vascular resistance and adverse birth outcomes, including LBW offspring and stillbirth (*10*). Collectively, those findings support the hypothesis that MIP dysregulates placental angiogenesis and vascular remodeling, resulting in placental insufficiency and poor birth outcomes. Here, we confirm and extend those observations and implicate MIP-induced changes in L-arginine–NO biosynthesis as a putative mediator of the altered angiogenesis observed. Of translational relevance, these changes can be corrected, at least in part, by L-arginine supplementation of malaria-infected dams. L-arginine treatment was associated with reduced placental expression of factors that destabilize blood vessels (*C5a*, *Ang-2*, and *Vegf-a*), as well as inflammatory cell adhesion molecules (*Icam-1*). Increased concentrations of these inflammatory factors and mediators of endothelial dysfunction have previously been linked with adverse birth outcomes in other conditions in pregnancy (*10*, *16*, *52*, *53*). Expression of *Ang-2*, *Tie-2*, and *Vegf-a* is increased under hypoxic conditions, which may also occur during MIP (*54*, *55*). We posit that the enhanced *Tie-2* expression we observed in L-arginine–supplemented dams promotes microvascular stability in the context of malaria-induced inflammation and vascular injury (*56*). We observed increased *Vegf-a* expression in the malaria-infected nonsupplemented dams, which was reduced with L-arginine supplementation. Together, these results are consistent with the hypothesis that L-arginine supplementation improves birth outcomes by reducing the expression of proinflammatory factors and by normalizing angiogenic processes and promoting placental function and fetal growth.

To link the observed L-arginine–related changes in inflammatory and angiogenic factors to a functional vascular correlate, we used micro-CT to visualize the impact of dietary supplementation on placental vascular structure and development. Consistent with previous studies, malaria infection was associated with altered vascular branching in the smaller vessels (*10*). Abnormal placental vascular development has previously been linked to poor birth outcomes, including fetal growth restriction and pre-eclampsia (*57*, *58*). Here, L-arginine supplementation in malaria-infected dams was associated with an increase in the total number of vessel segments, especially in smal L-diameter vessels (<50 µ m). These small terminal capillaries are the primary sites of vascular remodeling later in pregnancy (*58*) and therefore represent a biologically relevant site of action for the L-arginine–NO pathway. Collectively, the results suggest that L-arginine supplementation contributes to increased fetal weight and viability via expansion of the vascular network of the placenta, allowing for increased blood volume and surface area for nutrient exchange. In a previous preclinical study, MIP was associated with increased arterial resistance and poor birth outcomes, which were reversed by disruption of C5a signaling, and we report similar results here with L-arginine supplementation (*10*). However, L-arginine dietary supplementation represents a more feasible, safe, inexpensive, and acceptable intervention strategy for pregnancy compared to biologics for C5 blockade (*59*).

Altered angiogenesis may represent a common pathway of injury resulting in adverse birth outcomes associated with multiple pathological conditions in pregnancy, including pre-eclampsia, and L-arginine supplementation during pregnancy may improve birth outcomes in high-risk women (*16*, *17*, *46*). Several lines of evidence support this hypothesis. In many malaria-endemic regions, malaria-induced reductions in L-arginine may be further compounded by the lack of dietary L-arginine intake (*60*). Most regions with high rates of poor birth outcomes also have high rates of malnutrition due, in part, to low daily protein intake and, therefore, low L-arginine intake (*39*, *61*). Low dietary intake of L-arginine has been linked to an increased risk of PTB in Tanzanian women (*61*). Moreover, a previous randomized trial used medical food to supplement L-arginine in the diet (*62*) and reported reduced incidence of pre-eclampsia in a high-risk cohort of women receiving L-arginine supplementation. Here, the beneficial impact of L-arginine supplementation was most marked in animals on an L-arginine–deficient diet, suggesting that L-arginine supplementation may be most efficacious in women in low-resource settings who are most vulnerable to malaria-associated adverse birth outcomes.

Although the mouse model can provide important mechanistic insights into the pathophysiology of MIP, it also has limitations. The model replicates important components of *P. falciparum* malaria infection in pregnancy, including the induction of an inflammatory response in the placenta, shared placental vascular development and placental pathology, and associated adverse birth outcomes including intrauterine growth restriction and decreased fetal viability. However, there are also differences, including higher parasitemia in the mouse model, which is not observed in multigravid clinical cohorts, and the lack of VAR2CSA-mediated blinding of parasitized erythrocytes in the placenta. Notably, the mouse model used in this study most closely models infection in nonimmune primigravid women, where higher parasite burdens and the greatest risk of adverse birth outcomes are observed. Moreover, although *Plasmodium berghei* adhesion in the placenta is not mediated by the same receptors as *P. falciparum*, binding and accumulation of parasitized erythrocytes in the placenta are observed (*41*).

In summary, we provide evidence supporting the role of L-arginine–NO biosynthesis in the pathophysiology of MIP. In a prospective study of women with MIP, alterations in this pathway were associated with adverse birth outcomes. We demonstrate that similar changes occur in a preclinical model of MIP and use this model to demonstrate that strategies to enhance L-arginine bioavailability improve birth outcomes, at least in part by reducing placental inflammation, regulating angiogenesis, and enhancing placental vascular development. We propose that interventions aimed at promoting regulated angiogenesis in the placenta may improve birth outcomes and reduce the global burden of MIP.

## MATERIALS AND METHODS

### Clinical cohort study design and ethics

The objective of the clinical study was to quantify plasma concentrations of L-arginine, ADMA, and SDMA in a cohort of pregnant women in association with malaria infection. Samples were collected as part of a multisite, open-label, two-arm, randomized superiority trial in southern Malawi (Pan African Clinical Trials Registry PACTR20110300280319 and ISRCTN Registry ISRCTN69800930), which took place between 2011 and 2013, as previously described (*40*). Briefly, eligibility criteria included HIV-negative women with an estimated gestational age between 16 and 28 weeks of gestation by ultrasound, last menstrual period (LMP), or both; hemoglobin >7 g/dl at baseline; a willingness to deliver in hospital; and not having received a dose of SP in pregnancy. Women were randomized to receive one of the following over the second and third trimester of pregnancy: (i) three or four doses of SP (IPTp-SP) or (ii) screening with malaria rapid diagnostic tests (RDT) (First Response Malaria pLDH/ HRP-2 Combo Test, Premier Medical Corporation Ltd.) and treatment of RDT-positive women with a standard 3-day course of DP (ISTp-DP; 40 mg/320 mg of tablets; Eurartesim, Sigma-Tau). We randomly selected 384 primigravidae for the assessment of L-arginine, SDMA, and ADMA provided they met the following inclusion criteria: live birth with known birth weight and singleton delivery. Of the 384 women included, 379 had an enrollment sample tested and 94 had multiple samples tested over pregnancy for longitudinal assessment of L-arginine, SDMA, and ADMA. Written informed consent was obtained for all study participants. This study was reviewed and approved by the Liverpool School of Tropical Medicine, the Malawian National Health Science Research Committee, and the University Health Network Research Ethics Committee.

### Sample size calculation for the clinical cohort study

Our primary endpoint for the human cohort study was the association between the arginine pathway and adverse birth outcomes in primigravidae. Using pilot data from the enrollment visit, we estimated a sample size of 323 women, assuming a mean difference in ADMA of 8 ng/ml and an SD of 19, with 20% of women expected to have an adverse birth outcome (β = 0.80, α = 0.05). In case the data were not normally distributed, we adjusted our sample size upward by 15% to generate a final minimum sample size of 372 women.

### Assessment of L-arginine, ADMA, and SDMA

EDTA plasma samples were tested for L-arginine, ADMA, or SDMA using high-pressure liquid chromatography electrospray tandem mass spectrometry, as described below. The coefficients of variation for arginine testing were 5.2% for L-arginine, 2.0% for SDMA, and 1.4% for ADMA. Concentrations of L-arginine, ADMA, and SDMA were quantified as nanograms per milliliter, and the ratios are expressed as L-arginine/ADMA, L-arginine/SDMA, and ADMA/SDMA (*63*, *64*). All samples were analyzed blinded to the malaria infection status of the participants.

### Statistical analysis of the clinical cohort

For the human study, relative risk was calculated using a log-binomial model, including all variables with *P* < 0.20 by bivariate analysis. In addition, treatment arm, maternal age, and malaria status at enrollment (by microscopy) were included in the model. To compare the association between markers of NO biosynthesis and nutritional status (maternal BMI, MUAC, and hemoglobin), we used linear regression, adjusting for maternal age and gestational age at enrollment. For longitudinal analysis, we used linear mixed-effects modeling with the lme4 (*65*) package in R (*66*) to evaluate the relationship between longitudinal ADMA concentrations and the SGA outcome. We first constructed a null model with six fixed effects: the linear effect of gestational age, maternal age, enrollment BMI, enrollment malaria status, socioeconomic status, and the interaction between gestational age and treatment arm. This interaction term adjusted for the possibility that the rate of change of ADMA was affected by either treatment. Using likelihood ratio tests, we then assessed whether adding SGA as a fixed effect significantly improved the model fit, followed by adding the interaction between SGA and gestational age (table S1). For random effects, all models included a by-participant intercept and a by-participant slope for the effect of gestational age. Biomarker concentrations were transformed using the natural logarithm to stabilize their variance. No deviation from homoscedasticity or normality was apparent on the residual plots. Similarly, but without adjusting for other covariates, linear mixed-effects (LME) models were used to assess the relationship between malaria status at enrollment (by microscopy and PCR) and gestational changes in ADMA, SDMA, and L-arginine concentrations.

### EMIP study design and animal use protocols

The objectives of the studies using the EMIP model were to examine the impact of L-arginine supplementation on in utero development (viability and weight) in malaria-infected dams, as well as the impact of L-arginine supplementation on placental vascular development. The EMIP model used in this study is a validated murine model of MIP, which replicates key pathogenic factors of human MIP (*41*). Female wild-type BALB/c mice between 6 and 8 weeks of age were mated with male wild-type BALB/c mice (8 to 9 weeks of age, obtained from the Jackson Laboratory). Naturally mated pregnant mice were infected on G13 with 106 *P. berghei* ANKA (PbA)–infected erythrocytes in RPMI 1640 (Gibco) via injection into the lateral tail vein. Control pregnant females were injected on G13 with RPMI 1640 alone. Thin blood smears were taken daily and stained with Giemsa stain (Protocol Hema3 Stain Set, Sigma-Aldrich) to monitor parasitemia. Investigators were not blinded to the experimental group during treatment because the investigators had to prepare the inoculum and L-arginine–supplemented water. However, investigators were blinded during sample processing and outcome assessment, including tissue collection (G19, assessment of weight and viability), processing of samples [placental tissue for reverse transcription PCR (RT-PCR) and serum for mass spectrometry], and assessment of vascular development by micro-CT. All experimental protocols were approved by the University Health Network Animal Care Committee and performed in accordance with current institutional regulations.

### Dietary L-Arginine supplementation

On the day of pairing, mice were randomly assigned to one of the following treatment groups: (i) vehicle control (regular drinking water) or (ii) 1.2% L-arginine in drinking water (L-arginine monohydrochloride A6969, Sigma-Aldrich). Mice received L-arginine–supplemented drinking water (or vehicle control) beginning before pregnancy and a minimum of 13 days before malaria infection (depending on what day they became pregnant after pairing). A dose of 1.2% was selected because it represents about twice the daily intake of L-arginine in regular chow (50 mg/day, assuming a daily intake of 3 to 5 g of chow with 1% L-arginine), based on the assumption that mice drink 5 to 6 ml of water per day (60 mg/day intake via supplemented water). There was no difference in the daily intake of water between dams receiving the vehicle control and L-arginine–supplemented water at a dose of 1.2% L-arginine. All mice received treatment via ad libitum access to bottled drinking water throughout pregnancy. All supplementation treatments were given in autoclaved water and water bottles.

### L-Arginine–deficient chow

Dams that received L-arginine–deficient chow were placed on a diet of exclusively deficient chow (Harlan Laboratories) beginning at G9 (confirmation of pregnancy) until tissue collection. Dams were kept on their regular chow (Harlan Teklad) diet until this time (G9) to minimize disruptions to their environment (change in diet) during pairing and early pregnancy. Mice were assigned to the treatment groups, as defined above.

### Tissue collection

The EMIP model followed the protocol outlined above. Dams were sacrificed at G19 using carbon dioxide inhalation, yolk sacs were dissected from uteri, fetuses were removed and weighed, and placentas were snap-frozen and stored at −80°C until analysis. Fetal viability was determined by assessing pedal withdrawal reflex. Nonviable fetuses (lacking the pedal withdrawal reflex) were considered stillbirths. All fetuses were weighed at this time. RNA extraction was performed on snap-frozen fetal placenta tissue collected at G19. Serum from mice was collected from cardiac punch and stored at −80°C until analysis.

### Placenta transcript analysis

Only placentas collected from viable fetuses were used in the transcript analysis. Tissue was homogenized in TRIzol (0.5 ml/100 mg tissue; Invitrogen) according to the manufacturer’s protocol, and RNA was extracted. Extracted RNA (2 µg per sample) was then treated with DNase (deoxyribonuclease) I (Ambion) and reverse-transcribed to complementary DNA (cDNA) with SuperScript III (Invitrogen) in the presence of oligo(dT)18 primers (primer sequences in table S5) (Fermentas). Residual RNA was degraded with RNase (ribonuclease) H (Invitrogen). Sample cDNA was amplified in triplicate with SYBR Green Master mix (Roche) in the presence of forward and reverse primers (1 µ M both) in a Light Cycler 480 (Roche). Transcript number was calculated on the basis of *C*t (cycle threshold) compared to the standard curve of mouse genomic DNA included on each plate by Light Cycler 480 software (Roche) and was normalized to the geometric average of the expression of the housekeeping genes *Gapdh* and *Hrpt*.

### High-pressure liquid chromatography–electrospray tandem mass spectrometry

Concentrations of L-arginine, ADMA, and SDMA were assayed by mass spectrometry, as previously described (*67*). Briefly, the chromatographic conditions included a 125 × 3 mm Nucleosil 100-5 silica column with a 4 × 2 mm silica filter insert. Mobile phase A consisted of 1 liter of water mixed with 0.25 ml of trifluoroacetic acid and 10 ml of propionic acid. Mobile phase B consisted of 1 liter of acetonitrile mixed with 0.25 ml of trifluoroacetic acid and 10 ml of propionic acid. Isocratic elution with one part mobile phase A and nine parts mobile phase B was delivered at a flow rate of 0.5 ml/min at a temperature of 30°C. Samples were prepared with 60 µl of serum and 20 µl of the respective internal standard. Samples (10 µl) were injected automatically, and the electrospray ion source run time duration was 3 to 6.5 min under the following conditions: 32 (arbitrary units); auxiliary gas, 20 (arbitrary units); needle voltage, +4.5 kV; capillary temperature, 300°C.

### Placental micro-CT scans

Detailed methods for preparing the fetoplacental vasculature for micro-CT imaging have been described previously (*68*). Briefly, uteri were extracted from dams at G18 and anesthetized via hypothermia [immersion in ice-cold phosphate-buffered saline (PBS)]. Each individual fetus was then extracted from the uterus while maintaining the vascular connection to the placenta. The embryo was briefly resuscitated via immersion in warm PBS to resume blood circulation. Embryos that could not be resuscitated were not perfused and were removed from the study. A catheter was then inserted into the umbilical artery, and the fetus was perfused with saline [with heparin (100 U/ml)], followed by radiopaque silicone rubber contrast agent (Microfil, Flow Technology). After perfusion, specimens were postfixed with 10% formalin and imaged using micro-CT. Specimens were scanned at 7.1 µm resolution for 1 hour using a Bruker SkyScan 1172 high-resolution micro-CT scanner. A total of 996 views were acquired via 180° rotation with an x-ray source at 54 kVp (kilovolt peak) and 185 µA. Three-dimensional micro-CT data were reconstructed using SkyScan NRecon software. The structure of the vasculature was identified automatically using a segmentation algorithm, as previously described in detail (*69*). The leaves of the vascular tree were pruned to 0.035 mm (threshold diameter) to improve data consistency. Analysis was performed on wild-type [unexposed (*n* = 7) and malaria-exposed (*n* = 8)] offspring of control (nonsupplemented) dams and unexposed (*n* = 7) and malaria-exposed (*n* = 7) offspring of L-arginine–supplemented dams. Each group contained a minimum of three dams per group and one to three specimens per litter.

### Statistical analyses of EMIP-based studies

Statistical analysis was performed using Stata v14 (StataCorp), R v3.2.1 (R Core Team, 2015, R Foundation for Statistical Computing), and GraphPad Prism v6 (GraphPad Software Inc.). Student’s *t* test, oneway analysis of variance (ANOVA) (nonparametric Kruskal-Wallis, *P* < 0.05), post test (Tukey test), independent samples *t* test, χ^2^ test, and relative risk were used to examine the statistical significance of differences between experimental groups. Analysis of the cumulative distribution of vessel diameters for each placenta was fit with a natural spline with eight degrees of freedom. A two-way ANOVA was conducted to determine whether there was an effect of treatment group on the spline parameters. There was a significant interaction between spline coefficient and group (*P* < 0.001), and therefore, a post hoc analysis was performed to compare pairs of treatment groups. Post tests on all groups were conducted using Dunn’s multiple comparison test (*P* < 0.05).

## SUPPLEMENTARY MATERIALS

www.sciencetranslationalmedicine.org/cgi/content/full/10/431/eaan6007/DC1

Table S1. Linear mixed-effects modeling of longitudinal changes in ADMA and SGA.

Table S2. Dams’ peripheral parasitemia at G19 and litter size from all cohorts.

Table S3. Fetal and placental weight by treatment group.

Table S4. Fetal viability by treatment group.

Table S5. RT-PCR primer sequences (5′ to 3′).

## Data and materials availability

Maternal plasma samples are available from M.W., L.K.P., V.M., and F.O.t.K. under a material transfer agreement with the College of Medicine, University of Malawi, Blantyre, Malawi.
