# Supplementary material for: Malaria in pregnancy alters L-arginine bioavailability and placental vascular development
Source: Sci Transl Med. 2018 Mar 7;10(431):eaan6007. doi: 10.1126/scitranslmed.aan6007 (PMC6510298; doi:10.1126/scitranslmed.aan6007)
Supplement: Supplementary file 1 [file STM-10-eaan6007_S1.pdf]

Science Translational Medicine
AAAS

www.sciencetranslationalmedicine.org/cgi/content/full/10/431/eaan6007/DC1

Supplementary Materials for

Malaria in pregnancy alters L-arginine bioavailability and placental vascular development

Chloe R. McDonald, Lindsay S. Cahill, Joel L. Gamble, Robyn Elphinstone, Lisa M. Gazdzinski, Kathleen J. Y. Zhong, Adrienne C. Philson, Mwayiwawo Madanitsa, Linda Kalilani-Phiri, Victor Mwapasa, Feiko O. ter Kuile, John G. Sled, Andrea L. Conroy, Kevin C. Kain*

*Corresponding author. Email: kevin.kain@uhn.ca

Published 7 March 2018, *Sci. Transl. Med.* **10**, eaan6007 (2018)
DOI: 10.1126/scitranslmed.aan6007

This PDF file includes:

Table S1. Linear mixed-effects modeling of longitudinal changes in ADMA and SGA.
Table S2. Dams' peripheral parasitemia at G19 and litter size from all cohorts.
Table S3. Fetal and placental weight by treatment group.
Table S4. Fetal viability by treatment group.
Table S5. RT-PCR primer sequences (5′ to 3′).

Supplementary Table 1. Linear mixed-effects modeling of longitudinal changes in ADMA and SGA.

	Additive Model		Interaction Model	
	Beta	Std. Error	Beta	Std. Error
**Fixed effects**				
Intercept	-0.68348	0.198	-0.73756	0.201
SGA	0.07339	0.036	0.25998	0.093
Gestational age, weeks	0.00301	0.002	0.00513	0.002
Socioeconomic status, tertile				
1 (reference)				
2	0.00124	0.045	0.00158	0.045
3	-0.08904	0.039	-0.09118	0.039
Maternal age, years	-0.00500	0.010	-0.00496	0.010
Enrollment BMI, kg/m^2	-0.00184	0.006	-0.00215	0.006
Smear-positive malaria	0.00088	0.040	0.00301	0.040
Gestational age * Treatment arm	0.00087	0.001	0.00095	0.001
Gestational age * SGA			-0.00668	0.003
**Random effects**				
Number of participants	91		91	
Observations	298		298	
Likelihood Ratio Test			*Against null model*<br>$\chi^2(2) = 8.76$, $p < 0.02$<br><br>*Against additive model*<br>$\chi^2(1) = 4.62$, $p < 0.04$	

*Indicates an interaction term.

Supplementary Table 2. Dams' peripheral parasitemia at G19 and litter size from all cohorts.

		Unexposed			Malaria-exposed		
**Cohort Presented in Figure:**	**Treatment Group**	**Peripheral Parasitemia (%)**	**Litter Size**	**Number of Litters per Group**	**Peripheral Parasitemia (%)**	**Litter Size (Viable Offspring)**	**Number of Litters per Group**
**Figure 4**	Vehicle	0	5.8±0.47	22	51.8±7.36	5.6±1.31	26
	L-arginine	0	5.8±0.43	37	47.2±8.17	5.1±1.31	36
**Figure 8**	Vehicle	0	6.0±0.71	10	50.3±5.19	4.4±0.68	12
	L-arginine	0	5.8±0.63	10	49.4±7.28	4.8±1.54	11

Values are presented as mean ± SD. Results of one-way ANOVA and Tukey's post-test.

Supplementary Table 3. Fetal and placental weight by treatment group.

		Unexposed	Malaria Exposed
Fetal Weight (g)	Vehicle Control	1.084 ± 0.21 (127)	**0.806 ± 0.23 (206)***
	L-arginine-supplemented	1.103 ± 0.19 (150)	**0.835 ± 0.34 (183)***
Placental Weight (g)	Vehicle Control	0.127 ± 0.06 (127)	0.122 ± 0.06 (207)
	L-arginine-supplemented	0.119 ± 0.08 (149)	0.117 ± 0.067 (184)

Values are presented as mean ± SD (total number of pups per group). * $P < 0.05$ in an independent sample t-test

Supplementary Table 4. Fetal viability by treatment group.

		Vehicle Control	L-arginine Supplemented
Unexposed	% Viable	97.6% (144)	96% (144)
	% Non-viable	2.4% (3)	4% (6)
Malaria Exposed	% Viable	**49.8% (103)***	**61.4% (113)***
	% Non-viable	50.2% (104)	36.8% (71)

Values are expressed as percent of viable pups per study and total number of pups (n) per group. Results of one-way ANOVA and Tukey's post-test, *$P < 0.05$

Supplementary Table 5. RT-PCR primer sequences (5′ to 3′).

**Primer**	**Forward Sequence**	**Reverse Sequence**
Gapdh (Housekeeping)	TCAACAGCAACTCCCACTCTTCCA	TTGTCATTGAGAGCAATGCCAGCC
Hrpt (Housekeeping)	GCGCCCATGAAAGAAGTAAAA	TTCGATGACGTGCTCAAAAG
C5	TACCAATGCCAACCTGGTGAAAGG	TCTGCAGAACCTCTTTGCCCATGA
C5a receptor *(C5ar)*	ATCCTGCTGCTGGCTACCATTAGT	TCTGACACCAGATGGGCTTGAACA
Icam-1	CGGAAGGGAGCCAAGTAACTG	CGACGCCGCTCAGAAGAA
Tie-2	GCTTGCTCCTTTCTGGAACTGT	CGCCACCCAGAGGCAAT
Ang-1	CCTCTGGTGAATATTGGCTTGGGA	AGCATGTACTGCCTCTGACTGGTT
Ang-2	AGAGTACTGGCTGGGCAATGAGTT	TTCCCAGTCCTTCAGCTGGATCTT
Vegf-a	CATCTTCAAGCCGTCCTGTGT	ACTCCAGGGCTTCATCGTTACA
VEGF Receptor *(Vegfr)*	AGGTGAGCACTGCGGGCA	ATGAGTCCTTTAATGTTTGAC
